# Chromothripsis in myeloid malignancies

**DOI:** 10.1007/s00277-024-05814-9

**Published:** 2024-05-30

**Authors:** Chien-Yuan Chen

**Affiliations:** 1 Department of Internal Medicine, Division of Hematology, National Taiwan University Hospital, No. 7, Chung-Shan South Road, Taipei, 100 Taiwan; 2 Department of Pathology, Cytogenetic laboratory, National Taiwan University Hospital, Taipei, Taiwan

**Keywords:** Chromothripsis, Myeloid malignancy, Complex karyotype, ***TP53*** mutation

## Abstract

Chromothripsis refers to massive genomic rearrangements developed during a catastrophic event. In total acute myeloid leukemia (AML), the incidence of chromothripsis ranges from 0 to 6.6%, in cases of complex karyotype AML, the incidence of chromothripsis ranges from 27.3 to 100%, whereas in cases of AML with *TP53* mutations, the incidence ranges from 11.1 to 90%. For other types of malignancies, the incidence of chromothripsis also varies, from 0 to 10.5% in myelodysplastic syndrome to up to 61.5% in cases of myelodysplastic syndrome with *TP53* mutations.

Chromothripsis is typically associated with complex karyotypes and *TP53* mutations, and monosomal karyotypes are associated with the condition. *ERG* amplifications are frequently noted in cases of chromothripsis, whereas *MYC* amplifications are not. Moreover, *FLT3* and *NPM1* mutations are negatively associated with chromothripsis. Chromothripsis typically occurs in older patients with AML with low leukocyte counts and bone marrow blast counts. Rare cases of patients with chromothripsis who received intensive induction chemotherapy revealed low response rates and poor overall prognosis. Signal pathways in chromothripsis typically involve copy number gain and upregulation of oncogene gene sets that promote cancer growth and a concomitant copy number loss and downregulation of gene sets associated with tumor suppression functions.

Patients with chromothripsis showed a trend of lower complete remission rate and worse overall survival in myeloid malignancy. Large-scale studies are required to further elucidate the causes and treatments of the condition.

## Introduction

Chromothripsis refers to massive genomic rearrangements during a catastrophic event. The condition was first identified by Stephens et al., who used next-generation sequencing to identify chromothripsis in a man with chronic lymphocytic leukemia with 42 rearrangements involving the long arm of chromosome 4 [1]. They proposed that massive genomic rearrangements could occur during a single catastrophic event, causing cancer. Initially, chromothripsis was estimated to be present in only 2-3% of genomes of patients with cancer [1]. However, later studies have observed chromothripsis in the genomes of patients with several types of cancer [2–6] and patients with congenital anomalies [7–9]. Chromothripsis is thus one of the most common mechanisms involved in cancer development. The analysis of thousands of tumor samples has revealed that chromothripsis is prevalent in more than half of human cancer types [10, 11]. Chromothripsis occurs with a high frequency in liposarcomas, osteosarcomas, glioblastomas, and solid cancers such as esophageal cancer, lung cancer, and breast cancer. However, chromothripsis is relatively rare in myeloid malignancies [10].

Acute myeloid leukemia (AML) offers an excellent model to explore cancer mechanisms. The genetic evolution of AML is a dynamic process shaped by multiple cycles of mutation acquisition and clonal selection [12]. However, cases of AML developing suddenly without preceding history or abnormal laboratory results have also been reported. Chromothripsis in AML was first reported by Rausch et al. [13]. Chromothripsis has also been detected in other myeloid malignancies, but the incidence is heterogeneous (Table 1) [10, 13–28].

**Table 1:** Prevalence and diagnosis of chromothripsis in myeloid malignancies (AML, MDS, and MPN)

	Disease	Numbers of Patient	Diagnosis of chromothripsis	Reference
1	AML	8 of 17(47.1%) chromothripsis^+^ in TP53 mutant AML vs. 1 of 91(1.1%) chromothripsis^+^ in TP53 wild type AML	Ten switches between two or three copy-number states in SNP array	Rausch et al. Cell 2012 [13].
2	AML	22 of 56(39.3%) chromothripsis^+^ in complex karyotype AML vs. 0 of 255(0%) chromothripsis^+^ in non-complex karyotype AML		
3	AML	chromothripsis^+^ in 2 complex karyotype AML cases report	Multicolor FISH and SNP array	Mackinnon and Campbell. Cancer Genet 2014 [14].
4	AML	3 of 30 (10.0%) chromothripsis^+^ in therapy-related AML, 3 of 11 (27.3%) chromothripsis^+^ in with complex karyotype therapy-related AML 3 of 4 (75.0%) chromothripsis^+^ in TP53 mutant therapy-related AML	Ten switches between two or three copy number states in SNP array	Jacoby et al. Leukemia 2014 [15]
5	AML, MDS	7 of 67 (10.4%) chromothripsis^+^ in AML 7 of 17 (41.2%) chromothripsis^+^ in complex karyotype AML 2 of 19 (10.5%) chromothripsis^+^ in MDS 2 of 6 (33.3%) chromothripsis^+^ in complex karyotype MDS	Copy number states in SNP array	Kjeldsen E. Cancer genomics & proteomic 2015 [16].
6	MDS	3 of 301 (1.0%) chromothripsis^+^in high-risk MDS, including 61 MDS / MPN, and 240 MDS, all 3 patients with TP53 mutation, 2 of 3 were complex karyotype and one with non-informative karyotype,	ten changes in segmental copy number between two or three copy number states on an individual chromosome	Abáigar et al. PLoS one 2016 [17]
7	AML	18 of 49 (36.7%) chromothripsis^+^ in AML with marker chromosome 18 of 38 (47.4%) chromothripsis^+^ in complex karyotype AML 14 of 19 (73.7%) TP53 mutant in chromothripsis^+^ AML vs. 6 of 21 (28.6%) TP53 mutant in chromothripsis^−^ AML^−^	Ten switches between two or three copy-number states in SNP array	Bochtler et al. Blood 2017 [18]
8	AML	39 of 112(34.8%) chromothripsis^+^ in complex karyotype AML 33 of 39 (84.6%) TP53 mutant in chromothripsis^+^ complex karyotype AML vs. 39 of 73 (53.4%) TP53 mutant in chromothripsis^−^complex karyotype AML	Ten switches between two or three copy-number states in SNP array	Rücker et al. Haematologica. 2018 [19]
9	AML	26 of 395 (6.6%) chromothripsis^+^in AML 22 of 26 (84.6%) TP53 mutant in chromothripsis^+^ AML vs. 31 of 300 (10.3%) TP53 mutant in chromothripsis^−^ AML	SNP array, Korbel and Campbell’s criteria, three out of six criteria were satisfied	Fontana et al. Leukemia. 2018 [20]
10	AML	One complex karyotype AML case report	Clustering of breakpoints for chromosomes 19 and 22, regularity of Oscillating Copy-Number States for chromosome 11	Tolomeo et al. Cancer Genet. 2019 [21]
11	AML MDS MPN	0 of 8 (0%) AML, 0 of 1 (0%) MDS, and 0 of 7(0%) MPN Chromothripsis was not detected	whole-genome sequencing data	Cortés-Ciriano et al. Nat Genet. 2020 [10]
12	AML	9 of 26 (34.6%) chromothripsis^+^in AML-MRC (myelodysplasia-related changes) 9 of 17 (52.9%) chromothripsis^+^in complex karyotype AML-MRC	Array based. More than10 breakpoints with a minimum segment size of 10 kb.	Gao et al. Hum Pathol. 2020 [22]
13	AML	9 of 16 (56.3%) chromothripsis^+^in ERG amplification AML 9 of 15 (60.0%) chromothripsis^+^in complex karyotype ERG amplification AML 1 of 9 (11.1%) TP53 mutant in chromothripsis^+^in ERG amplification AML vs. 3 of 7(42.9%) TP53 mutant in chromothripsis^−^	equal to or greater than 10 alternating copy number changes	Lee et al. Genes Chromosomes Cancer. 2022 [23]
14	AML	11 of 11 (100%) complex karyotype with ERG amplification AML 9 of 10(90.0%) TP53 mutant in chromothripsis^+^ ERG amplification AML	SNP array based	Schandl et al. Cancer Genet. 2023 [24]
15	AML, MDS	Total 25 of 42 (59.5%) chromothripsis^+^ in TP53 mutates AML and MDS 17 of 29 (58.6%) chromothripsis^+^ in AML, 26 of 29 (90%) complex karyotype 8 of 13 (61.5%) chromothripsis^+^ in MDS, 13 of 13(100%) complex karyotype	Whole genome sequencing	Abel et al. Blood Adv. 2023 [25]
16	AML	6 of 11(54.5%) chromothripsis^+^ in complex karyotype AML	RNA sequencing and Hi-C 10 copy number changes on a single chromosome	Klever et al. Blood Adv. 2023 [26]
17	AML:	1 chromothripsis^+^complex karyotype AML	Cytogenetic, Multicolor-FISH, and Optical genome mapping	Coccaro et al. Genes. 2023 [27]
18	MPN	11/64 (17.2%) chromothripsis^+^ in blastic phase MPN 10/29 (34.5%) TP53 mutant / loss in chromothripsis^+^ blastic phase MPN		Brierley et al. bioRxiv. 2023 [28]

chromothripsis^+^: Positive for chromothripsis. chromothripsis^−^: Negative for chromothripsis

Chromothripsis is characterized by the aggressive clinical behavior of myelomas and melanomas and poor prognosis [2, 6]. Bochtler et al. reported chromothripsis detected or not which revealed no prognostic implication in AML patients with maker chromosome [18]. Fontana et al. reported that chromothripsis-positive patients had worse overall survival than patients with other cancer types [20]. However, the prognostic markers for AML are not well understood. The present study provides a review of the diagnostic criteria, incidence, cytogenetics, molecular findings, treatments, response, and prognosis of chromothripsis in myeloid malignancies.

### Mechanism

Crasta et al. identified a mechanism by which errors in mitotic chromosome segregation generate DNA breaks, leading to the formation of micronuclei [29]. The newly generated micronuclei undergo defective and asynchronous DNA replication, resulting in DNA damage and extensive chromosome fragmentation in the micronucleus, which subsequently reintegrate into the genome. This fragmentation and reintegration is one explanation for the development of chromothripsis in cancer and developmental disorders [29]. Zhang et al. employed a combination of live-cell imaging and single-cell genome sequencing to demonstrate that micronucleus formation can generate a spectrum of genomic rearrangements, some of which mirror the features of chromothripsis [30].

### Diagnosis of chromothripsis

Korbel and Campbell described conceptual criteria for the inference of chromothripsis by ruling out the alternative hypothesis that stepwise rearrangements of genetic material occurred, including (A) clustering of breakpoints, (B) regularly oscillating copy number states, (C) interspersed regions of lost heterozygosity, (D) prevalent rearrangements affecting a specific haplotype, (E) random DNA segment order and fragment joints, and (F) the ability to walk the derivative chromosome [31]. Because of the high cost of genomic sequencing, chromothripsis mostly identified by examining oscillating copy number variations in subsequent studies as Rausch et al. [13].

Rausch et al. inferred chromothripsis in cases in which at least ten switches between two or three copy number states were apparent on an individual chromosome, such as a sequence of the states ‘2’ and ‘1’ (‘2; 1; 2; 1; 2; 1; 2; 1; 2; 1; 2’), or ten switches between ‘2’ and a highly amplified state such as ‘30’ (‘2; 30; 2; 30; 2; 30; 2; 30; 2; 30; 2’) [13]. Most studies have suggested that chromothripsis follows the diagnostic rules of oscillating copy number variations [15–26]. Mackinnon and Campbell reported two AML cases of chromothripsis illustrating different aspects of this phenomenon from a cytogenetic perspective, and they employed fluorescence in situ hybridization (FISH) analyses such as multicolor FISH and multicolor banding to reveal chromothripsis [14].

For determining the organization of highly rearranged genomes, Mackinnon described strategies combining single-nucleotide polymorphism array data with various metaphase FISH methods [32]. Additionally, Coccaro et al. applied cytogenetic analysis, multicolor FISH, and optical genome mapping to fully characterize chromothripsis at high resolution [27]. However, although FISH and optical genome mapping could demonstrate chromothripsis, FISH studies require fresh samples, whereas optical genome mapping requires high-quality, long genomic DNA. Most retrospective studies cannot provide adequate specimens for research. Each method has benefit and limitation to detect chromothripsis.

### Incidence of chromothripsis in AML, MDS, and MPN

Chromothripsis has been reported in studies (Table 1) of patients with AML [10, 13–16, 18–27], myelodysplastic syndrome (MDS) [10, 16, 17, 25] and myeloproliferative neoplasms (MPNs) [10, 28]. The first study of chromothripsis in patients with AML was conducted by Rausch et al., who discovered that chromothripsis was associated with *TP53* mutations in medulloblastomas [13]. Chromothripsis is more commonly detected in AML patients with *TP53* mutations (8/17, 47.1%) than in AML patients with the *TP53* wild type (1/91, 1.1%), and it is more commonly detected in cases of complex karyotype AML (22/56, 39.3%) than in noncomplex karyotype AML cases (0/255, 0%) [13]. Fontana et al. reported 26 of 395 (6.6%) patients with AML exhibited chromothripsis [20]. The incidence of chromothripsis in total patients with AML is generally low (0-6.6%).

Similar conclusions have been reached in other studies: Chromothripsis is the most common in cases involving *TP53* mutations and complex karyotype AML and MDS [15–26] (Table 1). Only one study reported no chromothripsis in 16 patients with myeloid malignancy (8 patients with AML, 1 with MDS, and 7 with MPN); however, the karyotype and *TP53* mutation status of the patients in that study were not reported [10]. Studies have found that the incidence of chromothripsis ranged from 0 to 10.5% in patients with MDS, and chromothripsis was detected in 8 of 13 (61.5%) patients with *TP53* mutations, including 13 of 13 (100%) with complex karyotypes [10, 16, 17, 25]. By contrast, chromothripsis is rarely reported in patients with MPN [10, 28].

### Cytogenetic abnormalities, genetic mutations, and chromothripsis

Recurrent chromosomal abnormalities, such as t(8;21) and inv(16) in core binding factor leukemia or t(15;17) in acute promyelocytic leukemia, hold significant diagnostic and prognostic value for hematological malignancies. Cytogenetic abnormalities and genetic mutations associated with chromothripsis are shown in Table 2. 10–12% of all patients with AML have complex karyotypes, and the incidence of complex karyotypes increases with advanced age [33]. Complex karyotypes are associated with chromothripsis; the incidence of chromothripsis in patients with complex karyotype AML ranges from 27.3 to 100% [13, 15, 16, 18, 19, 22–26].

**Table 2:** Correlations between chromothripsis and cytogenetic and genetic abnormalities in myeloid malignancies

	Associated with Chromothripsis (reference)
**Cytogenetic**	
monosomy 5 / del(5q)	19,
monosomy 7/ del(7q)	19,
monosomy 12 / del(12p)	19,
monosomy 16 / del(16q)	19,
monosomy 17/ del 17p	19,
marker chromosome	18,
complex karyotype	13
monosomal karyotype	18
**Genetic**	
*TP53* status	13
*FLT3* status	20 (negative association)
*NPM1* status	20 (negative association)
*ERG* amplification	23

Rücker et al. reported chromothripsis in 39 of 112 patients with complex karyotype AML, and chromothripsis was associated with the chromosomal aberrations of − 5/del(5q), − 7/ del(7q), − 12/del(12p), − 16/del(16q), and − 17/del(17p) [19]. An abnormal chromosome of which no part can be identified through karyotyping is referred to as a marker chromosome. Bochtler et al. reported marker chromosomes in 165 of 1026 patients with noncore binding factor karyotype leukemia. Marker chromosomes were associated with chromothripsis, but not with the chromosomal aberrations of 5q, 7q, and 17p [18]. Additionally, Mackinnon and Campbell used multicolor FISH and cytogenetic banding analysis to diagram normal chromosomes before rearrangement, demonstrating that the normal chromosomes were broken in several segments before rejoining to form markers, rings, and derivative chromosomes in two cases of chromothripsis with complex karyotype AML [14].

Monosomal karyotypes are defined by the presence of a single autosomal monosomy (excluding the isolated loss of X or Y) in association with at least one additional autosomal monosomy or one structural chromosomal abnormality without core binding factor AML or acute promyelocytic leukemia [34]. Monosomal karyotypes are associated with poor prognoses in patients exhibiting complex karyotypes [34]. Monosomal karyotypes are associated with chromothripsis [18, 19].

*TP53* mutations or loss associated with chromothripsis have been reported in several studies involving patients with AML [13, 15, 17–20, 23–25]. The incidence of chromothripsis in patients with AML having *TP53* mutations ranges from 11.1 to 90% (Table 2) [13, 15, 17–20, 23–25]. *ERG* amplification is a secondary recurrent driver event in myeloid malignancies with complex karyotypes and *TP53* mutations [23]. *ERG* amplification is associated with chromothripsis [23, 24]. L’Abatte et al. employed high-resolution genomic methods to investigate amplicons containing *MYC* in 23 patients with AML and revealed no chromothripsis [35].

Fontana et al. reported that *FLT3* and *NPM1* mutations were negatively associated with chromothripsis [20]. *FLT3* and *NPM1* mutations are commonly observed in patients with normal karyotype AML, which is also negatively associated with chromothripsis. Finally, *IDH1/2, DNMT3A, CEBPA, RUNX1*, and *NRAS* mutations did not significantly differ between patients with and without chromothripsis [20].

### Clinical characteristics and chromothripsis

Bochtler et al. analyzed 49 patients with AML having marker chromosomes. Age, sex, prior MDS, leukocyte count, and lactate dehydrogenase (LDH) level did not differ between patients with and without chromothripsis [18].

Rücker et al. analyzed 39 AML patients with chromothripsis and 73 AML patients without chromothripsis [19]. Chromothripsis-positive patients were older (median age: 61 [range: 19–82] versus 56 [range: 21–76] years, *p* = 0.009) and had lower bone marrow blast counts (median 40% vs. 80%, *p* = 0.01). No differences were observed between patients in terms of sex, leukocyte count, platelet count, hemoglobin level, peripheral blood blast count, and serum LDH.

Fontana et al. analyzed 26 patients with AML with chromothripsis and 369 patients with AML without chromothripsis [20]. Significant differences were observed in age (67 vs. 60, *p* = 0.002) and leukocyte count (6,342 vs. 30,590, *p* = 0.04) between patients with chromothripsis and those without chromothripsis. Older patients had more complex karyotypes and *TP53* mutations, lower bone marrow blast counts, and lower leukocyte counts. The clinical characteristics of chromothripsis are consistent with the genetic and cytogenetic findings.

### Pathway-involved chromothripsis

Fontana et al. reported that, in pathway enrichment analysis of patients with AML with chromothripsis, DNA repair, E2F-mediated regulation of DNA replication, signaling pathways involving PI3K, phospholipid biosynthesis and metabolism, and various growth factor signaling pathways were in the top 1% of pathways enriched for amplification events, indicating a high association of these events with chromothripsis [20]. Additionally, CTLA4 inhibitory signaling, synthesis of phosphatidylinositol phosphate at the late endosome membrane, the Fanconi anemia pathway, and genes regulating G0, the early G1 phase, pre-NOTCH transcription, and translation were in the best 1% of pathways enriched for deletion events, indicating the high association of these deletion events with chromothripsis [20]. Furthermore, Klever reported copy number gain and the upregulation of gene sets, including genes amplified in patients with cancer having chromothripsis. Among other cancer-related genes, copy number loss and the downregulation of gene sets affect many genes with confirmed tumor suppressor functions [26].

### Treatment, response, and prognosis

Genetic and cytogenetic profiles have been thoroughly investigated for risk stratification in patients with AML. Mutational profiling can be used for risk stratification to inform prognostic and therapeutic decisions for patients with AML [36, 37]. The driver landscape of AML reveals distinct molecular subgroups reflecting discrete paths for the evolution of this cancer, enabling disease classification and prognostic stratification [38].

Fontana et al. reported that patients with chromothripsis had a poor response to induction chemotherapy, with only 3 of 10 patients with chromothripsis responding versus 152 of 229 patients without chromothripsis (*p* = 0.036). Additionally, patients with chromothripsis had worse overall survival (median of 120 days for patients with chromothripsis vs. 494 days for patients without chromothripsis, *p* < 0.001) [20].

Rücker et al. reported that chromothripsis was associated with a trend of lower complete remission rate (4/16 [25%] chromothripsis-positive vs. 21/45 [47%] chromothripsis-negative complex karyotype AML, *p* = 0.15) and thus predicted inferior survival. The estimated 2-year survival rates for chromothripsis-positive versus chromothripsis-negative patients were as follows: event-free survival, 0% versus 13% (log-rank; *p* < 0.001), relapse-free survival, 0% versus 34% (*p* < 0.008), and overall survival, 0% versus 28% (*p* < 0.001) [19].

Bochtler et al. reported cases from 2 large consecutive, prospective, randomized, multicenter intensive chemotherapy trials (AML96, AML2003) [18]. Marker chromosomes were detectable in 165 of 1026 (16.1%) patients with aberrant noncore binding factor karyotypes. Marker chromosomes were associated with poorer prognosis than other non-CBF aberrant karyotypes, leading to lower remission rates, inferior event-free survival, and inferior overall survival [18]. But chromothripsis detected or not which revealed no prognostic implication in a subgroup of AML patients with maker chromosome [18].

Chromothripsis is associated with poor prognosis in several malignancies [2, 6]. Only limited cases reported chromothripsis received intensive induction chemotherapy and showed fewer response rate and overall poor prognosis in AML [18–20]. Additionally, Fontana et al. reported 4 patients with chromothripsis receiving hypomethylating agents but did not report the outcomes [20]. Because most patients with chromothripsis have complex karyotypes and *TP53* mutations, hypomethylating agents with or without venetoclax can be considered as alternative treatment for patients with AML and chromothripsis.

### Further study

Most studies on patients with AML and chromothripsis follow the precedent of Rausch et al. and focus on complex karyotypes and *TP53* mutations [13, 15, 17–20, 23–25]. Complex karyotype AML is a heterogeneous entity. Although only 10–12% of patients have complex karyotypes, effective treatment approaches should be developed for these myeloid malignancies. Hypomethylating agents with or without venetoclax and hemopoietic stem cell transplants are promising alternative treatments for these patients. However, large-scale studies of the prognosis and treatment of chromothripsis should be conducted.

Noncomplex karyotype AML has been reported to be associated with chromothripsis [13, 18]. Thus, studies incorporating AML cases other than cases of complex karyotypes, MDS, and MPN are warranted to clarify the presentation of chromothripsis.

Double-minute karyotypes are associated with complex karyotypes characterized by frequent del(17p)/*TP53* mutations, micronuclei formation, myelodysplastic features, and dismal prognosis in patients with AML; these factors, in the aggregate, are highly suggestive of chromothripsis [39, 40]. Garsed et al. isolated and analyzed cancer-associated neochromosomes from liposarcomas at single-nucleotide resolution, revealing ring chromosomes in cases of chromothripsis involving chromosome 12 [41]. Additionally, derivative chromosomes have been suggested to be by-products of chromothripsis [14, 42]. Because cytogenetic studies in such cases enable prognostic stratification and diagnosis, establishing the link between chromosomal aberrations and chromothripsis may enable the creation of a prognostic scale for complex karyotype AML.

## Conclusions

Chromothripsis is commonly identified by examining oscillating copy number variations, and it is most common in cases of myeloid malignancies with complex karyotypes and *TP53* mutations. The incidence of chromothripsis ranges from 0 to 6.6% in total AML but ranges from 27 to 100% in cases of complex karyotypes and from 11.1 to 90% in cases of AML with *TP53* mutations. Chromothripsis is associated with monosomal karyotypes. *ERG* amplifications are frequently noted in cases of chromothripsis, but *MYC* amplifications are not. Moreover, *FLT3* and *NPM1* mutations are negatively associated with chromothripsis. Chromothripsis is characterized by low bone marrow blast counts and low leukocyte counts, and it most frequently occurs in older patients, which is consistent with genetic and cytogenetic findings. *TP53* mutations are associated with the low bone marrow blast counts and leukocyte counts commonly found in older patients, who typically have more complex karyotypes.

Patients with chromothripsis showed a trend of lower complete remission rate and worse overall survival in myeloid malignancy. The patient numbers are limited in current studies, and additional large-scale studies are required to clarify.

## Data Availability

No datasets were generated or analysed during the current study.
